# Reducing substance use and risky sexual behaviour among drug users in Durban, South Africa: Assessing the impact of community-level risk-reduction interventions

**DOI:** 10.1080/17290376.2017.1381640

**Published:** 2017-10-11

**Authors:** C.D.H. Parry, T. Carney, P. Petersen Williams

**Affiliations:** ^a^ PhD Psychology, is the Director of the Alcohol, Tobacco and Other Drug Research Unit at the South African Medical Research Council, Cape Town, South Africa; ^b^ Extraordinary Professor at the Department of Psychiatry, University of Stellenbosch, Tygerberg, South Africa; ^c^ PhD Psychiatry & Mental Health, is a Senior Scientist at the Alcohol, Tobacco and Other Drug Research Unit, at the South African Medical Research Council, Cape Town, South Africa; ^d^ Honorary Lecturer at the Department of Psychiatry and Mental Health, University of Cape Town, Cape Town, South Africa; ^e^ PhD Public Health & Family Medicine, is a Senior Scientist at the Alcohol, Tobacco and Other Drug Research Unit, at the South African Medical Research Council, Cape Town, South Africa; ^f^ Honorary Research Associate at the Department of Psychiatry and Mental Health, University of Cape Town, Cape Town, South Africa

**Keywords:** HIV risk behaviours, alcohol and other drug use, interventions, community outreach, comportements à risque pour le VIH, consommation de l’alcool et autres drogues, intervention, sensibilisation communautaire

## Abstract

Alcohol and other drug (AOD) use is increasingly recognised as having a direct and indirect effect on the transmission of human immunodeficiency virus (HIV). However, there is evidence to suggest that drug- and sex-related HIV risk-reduction interventions targeted at drug users within drug treatment centres or via community outreach efforts can lead to positive health outcomes. This study aimed to test whether a community-level intervention aimed at AOD users has an impact on risky AOD use and sexual risk behaviour. In 2007, in collaboration with a local non-governmental organisation (NGO) in Durban, an initiative was begun to implement a number of harm reduction strategies for injection and non-injection drug users. The NGO recruited peer outreach workers who received intensive initial training, which was followed by six-monthly monitoring and evaluation of their performance. Participants had to be 16 years of age or older, and self-reported alcohol and/or drug users. Peer outreach workers completed a face-to-face baseline questionnaire with participants which recorded risk behaviours and a risk-reduction plan was developed with participants which consisted of reducing injection (if applicable) and non-injection drug use and sex-related risks. Other components of the intervention included distribution of condoms, risk-reduction counselling, expanded access to HIV Testing Services, HIV/sexually transmitted infection care and treatment, and referrals to substance abuse treatment and social services. At follow-up, the baseline questionnaire was completed again and participants were also asked the frequency of reducing identified risk behaviours. Baseline information was collected from 138 drug users recruited into the study through community-based outreach, and who were subsequently followed up between 2010 and 2012. No injection drug users were reached. The data presented here are for first contact (baseline) and the final follow-up contact with the participants. There were no decreases in drug use practices such as use of cannabis, heroin, cocaine and Ecstasy after the intervention with drug users; however, there was a significant reduction in alcohol use following the intervention. While there was a substantial increase in the proportion of participants using drugs daily as opposed to more often, the reduction in the frequency of drug use was not statistically significant. Following the intervention, drug users had significantly fewer sexual partners, but there were no significant differences following the intervention with regard to frequency of sex or use of condoms. Substance use in general and during sex was, however, decreased. While the findings were mixed, the study shows that it is possible to provide HIV risk-reduction services to a population of substance users who are less likely to receive services through community outreach, and provide risk-reduction information, condoms and condom demonstration and other services. More intensive interventions might be needed to have a substantial impact on substance use and substance use-related HIV risk behaviours.

## Résumé

La consommation d’alcool et des autres drogues (AAD) est de plus en plus reconnue comme ayant un effet direct et indirect sur la transmission du virus d’immunodéficience humaine (VIH). Toutefois, il existe des preuves pour suggérer que les interventions de réduction du risque du VIH lié aux drogues et à l’activité sexuelle, ciblant les utilisateurs de drogues dans les centres de traitement de la toxicomanie, ou par des efforts de sensibilisation communautaires, peuvent avoir des effets bénéfiques pour la santé. La présente étude avait pour but de tester si une intervention au niveau communautaire visant les consommateurs d’AAD, a un impact sur consommation d’AAD ainsi que les comportements sexuels a risque. En 2007, en collaboration avec une organisation non-gouvernementale (ONG) locale à Durban, il fût entrepris de mettre en œuvre un certain nombre de stratégies de réduction du risque au bénéfice des utilisateurs de drogues injectables et non-injectables (UDIs/UDNIs). L’ONG a recruté des pairs agents communautaires qui ont reçu une formation intensive initiale, suivie d’un monitorage et de l’évaluation semestrielle de leur performance. Les participants étaient âgés d’au moins 16 ans, et auto-déclarés consommateurs d’alcool et/ou utilisateur de drogues. Les pairs agents communautaires ont rempli un questionnaire de base au cours des rencontres en face-à-face avec les participants, portant sur les comportements à risque. Le plan de réduction du risque était ensuite élaboré avec les participants et consistait en la réduction de l’usage des drogues injectables (le cas échéant) et non-injectables, et des comportements sexuels a risque. Les autres composantes de l’intervention incluaient la distribution des préservatifs, les conseils sur la réduction du risque, l’expansion de l’accès aux services de dépistage du VIH, au traitement et à la prise en charge du VIH et des affections sexuellement transmises, et les références aux services sociaux et de traitement de la toxicomanie. Lors du suivi le questionnaire de base fût rempli encore, et les participants enquêtés sur la fréquence de réduction des comportements à risque identifiés. Les informations de base ont été recueillies chez 138 utilisateurs de drogues recrutés dans l’étude grâce à la sensibilisation communautaire. Ils ont ensuite été suivis entre 2010 et 2012. Aucun utilisateur de drogues injectables n’a été recruté. Les données du premier contact (données de base) et celles du contact final lors du suivi des participants, sont présentées ici. Il n’y a pas eu de réduction des pratiques d’utilisation des drogues telles que le cannabis, l’héroïne, la cocaïne et l’Ectasie suite à l’intervention chez les utilisateurs de drogues; toutefois, il y avait une réduction significative de la consommation d’alcool suite a l’intervention. Bien qu’il y ait eu d’augmentation substantielle de la proportion de participants utilisant la drogue quotidiennement par opposition à plus souvent, la réduction de la fréquence d’utilisation de la drogue n’était pas statistiquement significative. Suite à l’intervention, les utilisateurs de drogue avaient significativement moins de partenaires sexuels, mais il n’y avait de différences significatives en ce qui concerne la fréquence des rapports sexuels ou d’utilisation du préservatif, suite à l’intervention. L’utilisation de la drogue en général et lors de l’acte sexuel avait cependant diminué. Bien que les résultats fussent mêlés, cette étude montre qu’il est possible de procurer de services de réduction du risque du VIH à une population d’utilisateurs de la drogue, qui est moins susceptible de recevoir de tels services par la sensibilisation communautaire; et de délivrer les conseils sur la réduction du risque, les préservatifs et démonstrations sur l’usage des préservatifs, et bien d’autres services. Des interventions plus intensives sont peut-être nécessaires ou avoir un impact substantiel sur l’usage des drogues les comportements à risque pour le VIH liés à l’usage des drogues.

## Background

Approximately 7 million people in South Africa are infected with the human immunodeficiency virus (HIV), accounting for 19.2% of adults aged 15–49 (The Joint United Nations Programme on HIV/AIDS [UNAIDS], 2017). In addition to the HIV epidemic gripping South Africa, the country has also experienced changes in drug consumption following its transition to democracy in 1994 and a rapid increase in the transshipment of a wide variety of drugs through South Africa and re-entry into the international drug-trafficking market. From the late 1990s and 2000s, this has led to an increased consumption of a broad range of drugs previously unavailable (Parry & Pithey, 2006; UNODC, 2005). KwaZulu-Natal is the province with the highest estimated HIV prevalence at 16.9% (Shisana et al., 2014), as well as known high levels of alcohol and other drug (AOD) use (Dada et al., 2017).

AOD use is increasingly recognised as playing a direct and indirect role in the transmission of HIV. This occurs directly, through the sharing of injection drug use paraphernalia and also indirectly, as AOD use can reduce inhibitions and impair judgement thus impacting on risky sexual encounters such as having multiple sexual partners and increased length of sexual encounters (Rosengard, Anderson, & Stein, 2004; Semple, Patterson, & Grant, 2004). Additionally, various studies have demonstrated inconsistent condom use among drug-using populations (Abdool, Sulliman, & Dhanoo, 2006; Dewing, Pluddemann, Myers, & Parry, 2006; McCurdy, Williams, Kilonzo, Ross, & Leshabari, 2005). Locally, studies have also demonstrated the impact of drug use on risky sexual behaviour where drug use facilitates high-risk sexual behaviour such as having multiple partners and inconsistent condom use (Parry, Carney, Petersen, Dewing, & Needle, 2008, 2009). Few studies have investigated HIV prevalence among drug users in South Africa. A report indicated the HIV prevalence among drug users in this country to be between 5.4% and 35% depending on the sub-population studied (e.g. injection drug users (IDUs) and non-injection drug users (NIDUs)) and other factors (Scheibe, Brown, Duby, & Bekker, 2011). They also concluded that further research is needed to better quantify the extent to which drug use impacts HIV status.

There is evidence to suggest that drug- and sex-related HIV risk-reduction interventions for drug users can lead to positive health outcomes (Copenhaver, Lee, & Margolin, 2007). Behavioural interventions targeting HIV risk behaviours among HIV positive and negative people have been recommended as an effective measure to minimise HIV transmission attributable to AOD use (Samet, Walley, & Bridden, 2007). In particular, Parry et al. (2009), following a rapid assessment of injecting and non-injecting drug users in three cities in South Africa, recommended that HIV and drug prevention and treatment efforts should be made more accessible and be better integrated with other services, and that community-based outreach efforts among those populations is needed to address HIV and drug risk. Community-based outreach programmes to reduce the spread of HIV/AIDS among drug users have been shown to be feasible in other African countries such as Kenya (Deveau, Levine, & Beckerleg, 2006), and with female commercial sex workers (CSWs) in Durban and with men-who-have sex with men (MSM) in Pretoria, South Africa (Carney, Petersen Williams, & Parry, 2016; Petersen Williams, Carney, & Parry, 2016). The latter projects involved creating partnerships between public health researchers and NGOs, and the employment by the NGOs of community-based peer outreach workers. In Brazil, the utility of involving community health workers (CHWs) in primary care and serving as a liaison between community members and medical providers and researchers has been researched. It has been found that collaborative experiences between different role players can facilitate the timely sharing of research findings with practitioners working on the ground and a more integrated approach can improve the quality of care provided to persons in need (Pinto, Wall, Yu, Penido, & Schmidt, 2012). The research conducted in Brazil has also shown how CHWs through empathetic communication and perseverance are able to promote patient health behaviours (Pinto, da Silva, & Soriano, 2012) and how certain providers are more willing to adopt evidence-based practices and respond positively to scientific input than others (Pinto, Yu, Spector, Gorroochurn, & McCarty, 2010).

Despite the recognition of the role of AOD use on HIV transmission, there is limited availability of HIV risk-reduction services within South African substance abuse facilities (Myers, 2010). Given the association between HIV risk behaviour and drug use, Myers (2010) argues that coupled with South Africa's high HIV prevalence rate, a strong case can be made for upscaling efforts to provide integrated HIV risk-reduction and substance abuse treatment services. Furthermore, although the drafting of South Africa's Third National Drug Master Plan gave prominence to the need to address drug abuse as part of broader HIV prevention efforts (Department of Social Development, 2013), interventions targeting this population group to address drug use and sexual risk behaviour in South Africa are scarce. The aim of this study was to test whether a community-level intervention aimed at AOD users has an impact on risky AOD use and sexual risk behaviour.

## Methods

In 2007, in collaboration with a local non-governmental organisation (NGO) (the South African National Council on Alcoholism and Drug Dependence – SANCA) in Durban, an initiative was begun to implement a number of harm reduction strategies for IDUs and NIDUs. The NGO was selected based on its expertise and extensive experience working with the specific target population. Between 2010 and 2012, a formal evaluation was undertaken of the extent of behaviour change that had been effected by the intervention among drug users who were accessed through outreach during that time and who were followed up at least once.

### Participants and data collection

The intervention was conducted in various neighbourhoods in the eThekweni Metropolitan Municipality (Durban), South Africa, an area that comprises over three million inhabitants in both urban and rural communities. The estimated household size is 3.4 and over 30% of the population is unemployed (Statistics South Africa, 2013). A number of locations were targeted including streets in residential and industrial areas, and hotspots where drug users are known to frequent, such as shelters and community-based organisations. In addition, it became apparent after discussions with organisations in their network that it was important to consider reaching the target population through other institutions such as further education training colleges and spiritual establishments.

To be included in the study, participants had to be 16 years of age or older, and self-reported alcohol and/or drug users. At the time of project conception, Department of Health guidelines which now prohibit the inclusion of 16- and 17-year-olds without parental consent had not been established and participants of this age were able to sign informed consent as per ethics approval. The quantity of alcohol or drugs used by the participants was not used as an exclusion criterion, as long as they self-reported substance use in the past 90 days. Participants who were eligible for inclusion and willing to participate signed an informed consent form. Peer outreach workers completed a face-to-face baseline questionnaire with participants, which recorded risk behaviours and a risk-reduction plan was developed with each drug user which consisted of IDU/NIDU-related risks, sex-related risks and HIV testing. Risk behaviours recorded included the use of different kinds of drugs (alcohol, cannabis, heroin, Ecstasy, cocaine, methamphetamine, inhalants, methcathinone and over-the-counter or prescription drugs) in the past 90 days, frequency of use of drugs, number of sex partners (male and female), the number of times they had engaged in different kinds of sexual behaviours (vaginal, anal and oral sex) in the past 90 days, condom use during different kinds of sex acts (vaginal sex, receptive anal sex, insertive anal sex and oral sex) and whether they had traded sex for money during the past 90 days. Intervals between follow-up appointments were not specified, but in some instances, outreach workers made appointments for follow-up at a time that suited the participants, and in other instances, follow-up appointments occurred spontaneously when outreach workers met up with participants at various events or social gatherings. At follow-up, the same questionnaire that had been administered at baseline was completed again. Participants were also asked how often they had engaged in each of a number of listed risk-reduction behaviours and whether they had been tested for HIV, had asked their partner to test for HIV or encouraged a friend to be tested. Behaviour change was thus self-reported and not observed. Ethics approval for conducting the study was granted by the Health Research Committee of the University of Stellenbosch (N08/07/184).

### Intervention

SANCA staff received training over five days on an intervention that was based on a local adaptation of the World Health Organization's *Training guide for HIV prevention outreach to injecting drug users* (Burrows, 2004). A local adaptation lessened the focus on injection drug use-related behaviours and increased the focus on substance-related sexual HIV-risk behaviour. In addition, the adapted manual emphasised drugs commonly used in South Africa. Furthermore, training was provided to SANCA staff throughout the project (via six-monthly or more frequent visits) by staff from the South African Medical Research Council (SAMRC). Between 2010 and 2012, SANCA recruited 20 peer outreach workers on a volunteer basis and they were paid a modest stipend. Project coordinators were appointed within the organisation to ensure the smooth running of the project and acted as the liaison between the SANCA and the SAMRC project manager. The intervention included the following: administering a short baseline questionnaire to find out about individual risk behaviours (such as types of substances used, frequency of use and sexual activities). They then developed a risk-reduction plan together with the client, which consisted of non-injection drug use (NIDU) related risks, sex-related risks and plans for HIV counselling and testing (HCT). The intervention session which was then offered to the clients included education about HIV, condom demonstration and the provision of referrals as needed.

At the follow-up appointment, both the questionnaire and risk-reduction plan were discussed again with the client to assess behaviour change and revise risk-reduction plans.

Intervention practices were monitored on a monthly basis and evaluated bi-annually. This was done through observations by the project coordinators who rated outreach workers’ performance in terms of provision of basic HIV information, discussion of a risk-reduction plan, referral to HCT, condom demonstrations and provision of relevant referrals. On a six-monthly basis, a sample of drug-using participants was also provided with a questionnaire to assess what interventions they had been offered and about their experience of the intervention process, i.e. did they find it a positive experience? The outcomes of these evaluations by project coordinators and drug-using participants were used to inform the training of the outreach workers and HCT counsellors. The HCT outcomes were not disclosed to the SAMRC staff but were used by SANCA to make referrals to care and treatment where necessary.

### Data analysis

Demographic information was collected at baseline, and descriptive statistics were provided on these variables. Data on substance use and sexual risk behaviour were collected at baseline and at each follow-up appointment. We assessed the normality of the data collected using the Shapiro–Wilks test of normality, and the results indicated that the data were non-normally distributed (*p* < 0.001). Therefore, we conducted bivariate analyses with continuous data (number of sex partners, number of times engaged in sex, number of times had unprotected sex, number of times traded sex, number of times had sex while under the influence of substances). The Wilcoxon Signed Rank Test was used to determine whether the difference from baseline to follow-up was statistically significant. For categorical data, we assessed the differences in proportions (types of substances used, types of substances used during sex) and used the chi-square tests of association. All statistics were analysed using 95% confidence intervals. IBM Statistical Package for the Social Sciences (SPSS) version 21 was used for data analyses.

## Results

### Sample characteristics

In total, baseline information was collected from 138 drug users recruited into the study through community-based outreach, whose substance abuse and HIV risk profile was assessed and who then received an intervention and were subsequently followed up between 2010 and 2012. Of these, 44.2% had one repeat contact, 34.8% had two repeat contacts, 10.9% had three repeat contacts, 1.4% had four repeat contacts, 3.6% had five repeat contacts and 0.7% had between six and eight repeat contacts with outreach workers. The data presented here are for first contact (baseline) and the final follow-up contact with the particular client. The median age was 30 years (IQR: 17–54) and drug users had a median of 11 years of formal education (IQR: 0–24). Almost half of the drug users reached were employed (49.6%) and had a partner (69.6%) (Table 1). All participants were NIDUs, as no IDUs were identified through the outreach and recruitment process.

**Table 1.** Participant demographic characteristics (*N* = 138) community-level risk-reduction intervention, KwaZulu-Natal Province, South Africa, 2010–2012.

Demographic variables	*n*	%
**Gender**		
Male	125	90.6
Female	13	9.4
**Occupation**		
Unemployed	68	49.6
Employed	46	33.6
Student/pupil	4	2.9
Other	19	13.9
**Marital status**		
Single	30	22.2
Partner	94	69.6
Married – opposite sex	7	5.2
Married – same sex	1	0.7
Divorced/separated	1	0.7
Other	2	1.5

### Substance use

Table 2 outlines the proportion of drug users using each substance at baseline (time_1_) and last follow up (time_2_) and the difference between these two times. Substances which were used by less than 5% of the sample were excluded from the table. There were only statistically significant reductions in the proportion of drug users using alcohol (*p* = 0.03) from time_1_ to time_2_. No significant differences were observed over time for cannabis, cocaine, heroin and Ecstasy use. There was also no significant change in the frequency of substance use (*p* = 0.08). At time_1_, 41.3% reported daily use of substances and 39.1% reported daily use at time_2_, while more drug users were using substances once a week or less often and fewer using two to six days a week (Table 2). The use of more than one substance (polydrug use) was recorded at time_1_ and time_2_. In total, 45.7% of drug users did not report any changes in the number of different substances used, 23.2% increased the number of different substances they used and 31.1% decreased the total number of different substances used over the follow-up period; however, this difference was not statistically significant (*p* = 0.172).

**Table 2.** Prevalence of substance use at Time_1_ and Time_2_ (*N* = 138 participants).

Substance	*N* (Time_1_)	*N* (Time_2_)	*z*	*p*
Alcohol	124	111	2.22 (0.02; 0.18)	0.026*
Cannabis	73	65	0.96 (−0.06; 0.18)	0.335
Cocaine	9	3	1.75 (−0.004; 0.09)	0.080
Heroin	13	14	−0.20 (−0.08; 0.06)	0.845
Ecstasy	7	4	0.93 (−0.02; 0.07)	0.351
Frequency of use	% Time_1_	% Time_2_**		0.078
Once a week or less	23.2	32.6		
2–6 days a week	35.5	26.1		
Daily	41.3	39.1		
Change in drug and alcohol	*N*	%		0.172
No change	63	45.7		
Increase in AODs by				
1	29	21.0		
2	3	2.2		
3	0	0.0		
Decrease in AODs by				
1	33	23.9		
2	8	5.8		
3	2	1.4		

*Significance level *p* < 0.05.

**3 (2.2%) missing.

### Sexual risk behaviour and substance use

Following the intervention, drug users had significantly fewer sex partners (*z* = −2.84; *p* = 0.005); however, there were no significant differences with regard to frequency of sex or use of condoms (Table 3). Table 4 shows that there were significant reductions in the frequency of times that participants had sex while using substances (*p* = 0.05), as well as the proportion of drug users using cannabis (*p* = 0.04) during sex from time_1_ to time_2_, while no significant differences were observed for alcohol, cocaine, heroin, Ecstasy and inhalant use during sex from time_1_ to time_2_. In total, 39.1% of the drug users did not report any changes in the number of different substances used during sex, 21.7% increased the number of different substances that they used during sex and 39.1% decreased the total number of different substances used during sex.

**Table 3.** Sexual risk behaviour reported at time_1_ and time_2_ (in the past 90 days).

Sexual risk	Median (range) Time_1_	Median (range) Time_2_	*z*	*p*
Number of sex partners	2 (0–36)	1 (0–12)	−2.839	0.005*
Number of male partners	0 (0–10)	0 (0–48)	−1.929	0.054
Number of female partners	1 (0–36)	1 (0–8)	−3.627	0.000*
Times had vaginal sex	15 (0–90)	15 (0–90)	−0.152	0.879
Times had receptive anal sex	0 (0–10)	0 (0–1)	−1.857	0.063
Times had insertive anal sex	0 (0–40)	0 (0–24)	−1.025	0.305
Times had oral sex	0 (0–41)	0 (0–30)	−1.389	0.165
Times used condoms for vaginal sex	2 (0–90)	0 (0–30)	−0.522	0.602
Times used condoms for receptive anal sex	0 (0–0)	0 (0–0)	0.000	1.000
Times used condoms for insertive anal sex	0 (0–0)	0 (0–0)	0.000	1.000
Times used condoms for oral sex	0 (0–16)	0 (0–1)	−1.761	0.078
Times traded sex for money	0 (0–80)	0 (0–80)	−2.105	0.035*

*Significance level *p* < 0.05.

**Table 4.** Sexual risk and substance use reported at time_1_ and time_2_ (during past 90 days).

Substance	*N* (Time_1_)	*N* (Time_2_)	*z*	*p*
Alcohol during sex	81	67	1.68 (−0.02; 0.22)	0.092
Cannabis during sex	80	63	2.04 (0.01; 0.24)	0.041*
Cocaine during sex	6	2	1.45 (−0.01; 0.07)	0.148
Heroin during sex	8	13	−1.13 (−0.10; 0.03)	0.259
Ecstasy during sex	5	4	0.33 (−0.04; 0.05)	0.743
Inhalants during sex	28	25	0.29 (−0.06; 0.08)	0.773
Frequency of use during sex	Median (IQR) Time_1_	Median (IQR) Time_2_	*z*	*p*
Times had sex while using drugs and alcohol	5 (0–80)	5 (0–90)	−1.99	0.046*
Change in drug and alcohol use while having sex	*N*	%		
No change	54	39.1		
Increase in AODs by				
1	26	18.8		
2	4	2.9		
3	0	0.0		
Decrease in AODs by				
1	40	29.0		
2	13	9.4		
3	1	0.7		

*Significance level *p* < 0.05.

## Discussion

Following this brief behavioural intervention, there were not any decreases in drug use practices such as use of cannabis, heroin, cocaine and Ecstasy; however, there was a significant reduction in alcohol use over time. Previous studies indicate that brief behavioural interventions such as this one have been successful in reducing the amount of alcohol used by substance-using participants even outside of treatment settings (McCambridge & Kypri, 2011; Moyer, Finney, Swearingen, & Vergun, 2002). In addition, the reduction of alcohol use among NIDUs is important because alcohol use has been associated indirectly with HIV through risky sex behaviours and alcohol is one of the substances targeted in behavioural interventions for drug users (Lan, Scott-Shedon, Carey, Johnson, & Carey, 2014; Samet et al., 2007).

While there was a substantial increase in the proportion of participants using drugs daily as opposed to more often following the intervention, the reduction in the frequency of drug use was not statistically significant (*p* = 0.08). Even if use *per se* for illicit drugs did not decrease, a reduction in the number of times substances are used in a given period of time is likely to reduce harms at both an individual and at a population level (Wechsberg et al., 2008). Following the provision of the intervention, risky sexual practices in general were not reduced. Among other findings, there was no significant change (increase) in condom use during sex following the intervention. This could possibly be due to the type of sexual partner that the participants had. For example, a recent systematic review that addressed condom use in sub-Saharan Africa and Asia found that there is not much evidence on the intervention impact on condom use in casual relationships and in primary partnerships, unless one partner was HIV positive, engaged in high-risk behaviour or were trying to avoid becoming pregnant (Foss, Hossain, Vickerman, & Watts, 2007). The only significant change was that there was a decrease in the number of sexual partners reported at the follow-up period, which again is likely to lead to a reduction in harms at both an individual and population level (Kalichman et al., 2008; Mah & Halperin, 2010).

Substance use in general and during sex was decreased following the delivery of the intervention. Although this does not directly speak to the amount of risk reduced, having sex while intoxicated has been found in previous South African studies to be associated with impaired judgement, and reduced condom use and other risky sex behaviours (Kalichman, Simbayi, & Cain, 2010; Parry & Pithey, 2006; Wechsberg et al., 2012). However, the only substance that was specifically mentioned as being used less during sex following the intervention was cannabis. Although cannabis has not traditionally been linked to sexual risk behaviour, there is some evidence that it is specifically related to high-risk behaviour (Carney, Petersen Williams, Pluddeman, & Parry, 2015; Hendershot, Magnan, & Bryan, 2010).

The study is one of the first conducted in South Africa with NIDUs that shows that it is possible to provide HIV risk-reduction services to this population of substance users who are less likely to receive services through community outreach, and provide risk-reduction information, condoms, condom demonstration and other services as suggested by the World Health Organization guidelines (Burrows, 2004). This is similar to the findings of Deveau et al. (2006) who implemented such a community-based HIV prevention intervention for injection drug use in other African countries. While such interventions have on occasion been provided to a broad range of drug users attending drug treatment centres (Copenhaver et al., 2007; Grella, Etheridge, Joshi, & Anglin, 2000; Lally et al., 2005), community outreach to reduce HIV risk has generally not been a feature of interventions aimed at drug users who are not CSWs, MSM or IDUs. While not specifically studied, in line with the findings of Pinto, da Silva, and Soriano (2012) and Pinto, Wall, et al. (2012), it is highly likely that our choice of peer outreach workers of similar age and race to most of the drug-using study participants as well as their empathetic communication and perseverance also played a role in facilitating some of the behaviour changes that were observed.

The results of this study also need to be interpreted carefully as there were a number of limitations. First, because of the exploratory nature of the research and the fact that we were seeking to get the NGO to implement new outreach activities and interventions which would be evaluated in terms of their outcomes rather than a more controlled experiment, the study did not include a control group. It is thus not possible to determine if the changes we observed as a result of the risk-reduction counselling and other interventions would have occurred spontaneously. The sample size was also limited due to the intensity of the outreach and follow-up activities and thus it is possible that real changes over time were not detected. Furthermore, drug users themselves self-reported their substance use, and no biological tests were conducted. While peer outreach workers established relationships with drug users which may have improved the validity of self-reporting, the confirmation of substance use by urinalysis may have added to the legitimacy of the study. The time between baseline and follow-up assessments with the outreach workers, and therefore between intervention and assessments, also varied between participants meaning that for some there was more of a recency effect of the intervention than for others. In addition, participants were only recruited from one municipality in KwaZulu-Natal. It is therefore uncertain if the findings of this study are generalisable to substance users in South Africa overall, especially as there are cultural nuances in alcohol and drug culture in different parts of the country. Furthermore, although the study aimed to target both IDUs and NIDUs, IDUs were not reached in this study. IDUs have a number of additional risks for HIV associated with their injecting use, such as sharing needles and re-using syringes (Reid, 2009; Samet et al., 2007), as found in previous South African studies conducted by the current authors (Parry et al., 2009).

Future studies could target IDUs specifically, to assess if community-based interventions could be implemented in South Africa as they have been in other African countries (Deveau et al., 2006). In addition, it would be advantageous for future research to have an increased sample size to increase the statistical power of the analyses and also to include a control group (possibly a group of drug users on a wait list) so as to also reduce the possibility of any changes being observed as a result of other factors that had not been accounted for, such as reduced availability of drugs in the area. Having some or all of the participants provide biological specimens (such as urine) would also be useful to verify self-reported drug use. Further research is also needed to investigate why there were only reductions in specific drugs in the current study. Given the intersectoral innovation underlying this research, that is, the linking of NGOs, community (peer outreach) workers and researchers (Pinto, da Silva, & Soriano, 2012), it would also be useful to better measure the dynamics going on between the various players (e.g. NGO and researchers, NGO and peer outreach workers, researchers and peer outreach workers, and peer outreach workers and drug users).

## Conclusion

Despite the above-mentioned limitations, the findings of the current study and previous studies demonstrate that interventions targeting NIDUs can reduce their substance use to a certain degree, but may not affect other risk behaviours among this population. In South Africa, limited attempts to bring HIV prevention, treatment and care interventions into services for drug users have been made, if these do exist they have been associated with a number of challenges such as finances, geographic access barriers and awareness of available services (Myers, 2010). This study shows that it is feasible and acceptable to promote such comprehensive services with substance users, but that further nuances, such as more intensive interventions, might be needed to have a substantial impact on substance use and substance use-related HIV risk behaviours.
